# Oncostatin M Modulation of Lipid Storage

**DOI:** 10.3390/biology4010151

**Published:** 2015-02-13

**Authors:** Carrie M. Elks, Jacqueline M. Stephens

**Affiliations:** 1 Adipocyte Biology Laboratory, Pennington Biomedical Research Center, 6400 Perkins Road, Baton Rouge, LA 70808, USA; E-Mail: carrie.elks@pbrc.edu; 2 Department of Biological Sciences, Louisiana State University, 202 Life Sciences Building, Baton Rouge, LA 70803, USA

**Keywords:** oncostatin M, gp130 cytokines, adipocyte, obesity, adipose tissue

## Abstract

Oncostatin M (OSM) is a cytokine belonging to the gp130 family, whose members serve pleiotropic functions. However, several actions of OSM are unique from those of other gp130 cytokines, and these actions may have critical roles in inflammatory mechanisms influencing several metabolic and biological functions of insulin-sensitive tissues. In this review, the actions of OSM in adipose tissue and liver are discussed, with an emphasis on lipid metabolism.

## 1. gp130 Cytokines

The interleukin (IL)-6 family is a group of structurally similar cytokines that includes IL-6, IL-11, IL-27, leukemia inhibitory factor (LIF), oncostatin M (OSM), ciliary neurotrophic factor, cardiotrophin-1 (CT-1), novel neurotrophin-1/B cell stimulating factor-3 or cardiotrophin-like cytokine, and neuropoietin (reviewed in [1]). Since all members of this cytokine family require glycoprotein 130 (gp130) as a common signal transducer in their receptor complexes, the IL-6 family members are referred to as the gp130 cytokines. The principal signal transduction pathway facilitating the response to gp130 cytokines is the Janus Kinase/Signal Transducer and Activator of Transcription (JAK/STAT) pathway, which primarily activates STAT3.

The gp130 cytokines regulate a diverse array of biological processes, including hematopoiesis, immune responses, inflammation, stem cell potency, cardiovascular pathophysiology, and neuronal survival (reviewed in [2]). Circulating levels of many gp130 cytokines, namely IL-6, CT-1, LIF, and OSM, have been observed in humans [3,4,5,6,7,8,9]. Though the actions of this cytokine family in modulating lipid metabolism and fat cell function have not been fully elucidated, some gp130 cytokines, including CNTF and IL-6, have been targeted as potential therapeutic strategies in the treatment of obesity [10]. Some gp130 cytokines can exert profound effects on adipose tissue (AT), body weight, and glucose and lipid metabolism in rodents and humans [11,12,13,14,15,16,17,18,19,20]. AT and its primary constituents, adipocytes, are highly responsive to gp130 cytokines [19,21,22,23,24,25]; however, the effects of these cytokines in regulating lipid accumulation have not been fully characterized.

## 2. Oncostatin M

OSM is a gp130 cytokine that shares substantial sequence identity with LIF [26]. LIF and OSM evolved by gene duplication relatively recently [27]. Though originally identified for its ability to inhibit cancer growth in humans [28], OSM can modulate a variety of biological processes. However, unlike other gp130 cytokines, OSM has its own specific receptor, OSMRβ, that heterodimerizes with gp130 [29] and mediates the majority of OSM effects. There is some evidence that OSM is the only gp130 cytokine with the unique ability to signal through two distinct receptor units—the gp130/LIF receptor (LIFR) complex [30] and the gp130/OSMRβ complex [29]. However, other studies have shown that murine OSM only signals through the gp130/OSMRβ receptor complex [31,32]. These results have been confirmed, and since there is some receptor cross-talk among gp130 cytokines [33], it is critical to note that that mouse OSM only results in the tyrosine phosphorylation of gp130 and OSMRβ and does not signal via LIFR [31,32,33]. Similarly, human OSM only activates gp130 and OSMRβ and not LIFR in human adipocytes.

OSM can regulate inflammatory responses and is produced by activated T cells and macrophages [28,34,35]. In murine models of chronic inflammatory diseases, such as rheumatoid arthritis (RA) and multiple sclerosis, OSM has been shown to suppress inflammation [36]. Conversely, elevated OSM expression is found in a variety of inflammatory diseases in humans, including RA and atherosclerosis [37,38,39]. OSM has important roles in liver development and regeneration [40,41,42], hepatic insulin resistance and steatosis [43], inflammation [44], and cardiomyocyte dedifferentiation and remodeling [45]. Nevertheless, the actions of OSM on metabolic functions have not been fully elucidated and recent studies suggest pro-inflammatory roles of OSM on adipocytes [46]. Importantly, AT *in vivo* is highly responsive to OSM as compared to other insulin-sensitive tissues [33]. Mice given acute (20 min) OSM injections had robust STAT phosphorylation in two white AT depots (epididymal and retroperitoneal). The response to OSM in brown AT, liver, and skeletal muscle was minimal compared to white AT [33]. It is also known that OSM can inhibit adipocyte differentiation *in vitro* [25,47]. Several gp130 cytokines can have differential effects on both adipogenesis and insulin-stimulated glucose uptake *in vitro* and *in vivo* [19,21,22,23,24,25]. However, the actions of OSM on insulin signaling, lipid metabolism and glucose uptake require further investigation.

## 3. OSM Modulation of Adipocyte Differentiation

There are many hormones that affect lipid storage by promoting and/or inhibiting adipocyte development (reviewed in [48]). In the recent past, it was speculated that inhibitors of adipocyte development may act as suitable anti-obesity therapeutics by attenuating fat cell mass. In obesity, AT expansion can be accompanied by adipocyte dysfunction and alterations in adipokine secretion that can contribute to the development of metabolic diseases (reviewed in [49]). Cytokines that inhibit adipogenesis, such as tumor necrosis factor alpha and interferon gamma, tend to have metabolically unfavorable effects such as the induction of insulin resistance (reviewed in [50]). These cytokines and other adipocyte differentiation inhibitors can block fat cell expansion, a condition that has been recognized as a causative factor for insulin resistance for over a decade [51].

Several groups have demonstrated that OSM inhibits adipocyte development *in vitro* [25,33]. Also mice that have a global deletion of OSMRβ have increased adiposity [52], supporting the notion that OSM acts to block adipogenesis and that lack of OSM signaling leads to increased AT expansion. There is also evidence to suggest that OSM treatment of mice results in weight loss accompanied by decreased fat mass and, therefore, less lipid accumulation [52,53]. However, the OSM doses used in these studies were very high (12.5 ng/g body weight, administered twice daily) [52,53] and may have caused illness in the animals. In humans with diseases such as cancer and periodontitis, OSM is present in circulation at values ranging from 20 pg/mL to 100 pg/mL [54,55], while OSM is minimally detectable in healthy individuals. Therefore, the doses used in these animal studies are at least three to four orders of magnitude above the highest reported circulating OSM value for a human, and the impact of these high doses is likely much greater in mice. Nonetheless, these independent studies strongly suggest that OSM inhibits adipocyte development both *in vitro* and *in vivo*.

## 4. OSM is an Adipokine

**Figure 1:**
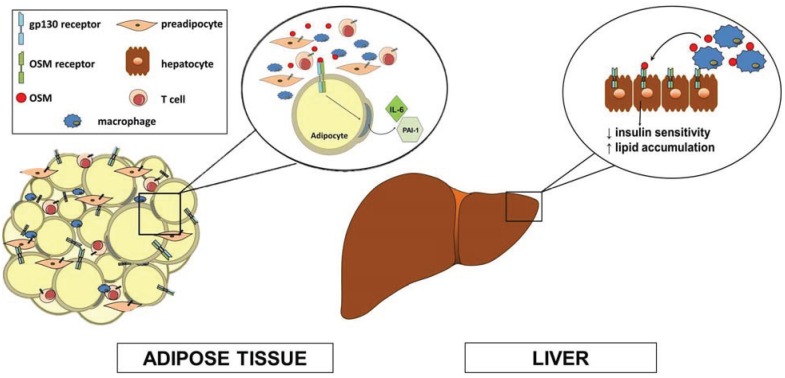
Oncostatin M (OSM) is produced in adipose tissue and liver immune cells and acts in a paracrine manner in these insulin-sensitive tissues. OSM is produced in T cells and macrophages that are found in adipose tissue and are elevated in conditions of obesity/insulin resistance. OSM receptors are present on preadipocytes and adipocytes, but not macrophages. It is well-known that OSM inhibits adipocyte differentiation. In addition, OSM acts on adipocytes to induce IL-6 and PAI-1 production. These abilities of OSM suggest that blocking OSM receptor activity in preadipocytes and adipocytes may be metabolically beneficial. In liver, OSM is produced in Kupffer cells (resident macrophages) and acts in a paracrine manner on hepatocytes to decrease insulin sensitivity and increase liver lipid accumulation.

An important AT function includes the production and secretion of factors (adipokines) that mediate whole body metabolism. Several adipokines can modulate physiological systems, as well as pathological conditions such as insulin resistance and inflammation (reviewed in [56]). AT is a well-known source of two gp130 cytokines, IL-6 [57,58] and CT-1 [7]. Previous studies have shown that murine adipocytes *in vitro* and white AT *in vivo* are responsive to OSM [33]. Hence, we examined OSM expression in mouse and human AT. We demonstrated that OSM mRNA and protein levels were elevated in AT of both *ob/ob* mice and high-fat diet (HFD)-fed B6 mice [46]; these rodent models are both used to study obesity and type 2 diabetes (T2DM). An analysis of human subcutaneous AT revealed that OSM levels correlated with body mass index (BMI), with substantial inductions of OSM protein levels seen in AT from subjects with BMIs of 40 or above [46]. These were the first studies to demonstrate that OSM mRNA and protein levels are elevated in obese/T2DM mice and humans. Interestingly, OSM is present in non-adipocyte cells of human AT. In mice, OSM is present in both CD11c+ and CD11c− macrophages that are F4/80+ and are found in AT of wild type and *ob/ob* mice [46]. OSMRβ is highly expressed in adipocytes, and OSM treatment of adipocytes induces IL-6 and PAI-1 expression [46,59]. In addition to positively correlating with body weight, increased OSM levels also positively correlate with insulin levels and are negatively associated with glucose disposal rates in humans [46]. Collectively, our data suggest that AT-derived OSM acts on preadipocytes and adipocytes (see Figure 1) and may modulate glucose and lipid metabolism and contribute to metabolic disease states.

## 5. Hepatic Effects of OSM

The hepatic functions of OSM are diverse and well documented [40,41,42,43,60,61,62,63,64]. A few years after the discovery of human OSM, its role as a potent inducer of the acute phase response in rat primary hepatocytes was identified [64]. Similar effects of mouse OSM were subsequently reported [65]. Evidence also suggests that hepcidin, a protein produced by the liver and a key regulator of iron metabolism, is an OSM target [60] and that OSM may play a role in anemia-associated disorders [60,62]. Interestingly, critical roles for OSM in fetal hepatic cell differentiation and hematopoiesis have also been reported [42]. Liver regeneration capability is impaired in OSMRβ knockout mice [41], and OSM has been shown to stimulate rat oval cell differentiation into hepatocytes, suggesting a role for OSM in the regenerative ability of hepatic tissue [63].

Further, OSM was found to up regulate the LDL receptor and rapidly increase phospholipid and diacylglycerol metabolism in HepG2 cells, highlighting a possible role for OSM in hepatic lipid metabolism [61]. Further support of this potential role is demonstrated in Kupffer cells, whose OSM production can inhibit the expression of several key enzymes of hepatic lipid metabolism [43]. Kupffer cell OSM has also been shown to attenuate insulin-stimulated Akt phosphorylation and glucokinase induction in hepatocytes, and is also up regulated in non-alcoholic steatohepatitis [43]. These results suggest overall that OSM produced by Kupffer cells may have a paracrine effect on hepatocytes to cause insulin resistance and lipid accumulation, and that these effects may contribute to systemic insulin resistance and metabolic syndrome. These results are consistent with observations of OSM being produced in AT macrophages (ATMs) and modulating pro-inflammatory genes in adipocytes (see Figure 1).

## 6. Metabolic Studies in OSMRβ Knockout Mice

Two recent studies from Komori and colleagues in OSMRβ-deficient mice suggest a metabolically protective role for OSM signaling [52,53]. However, it is necessary to interpret these studies with caution, as they were conducted in global OSMRβ KO mice. Since OSMRβ plays a known role in hematopoiesis and other biological functions beginning prior to birth [42], it is possible that many of the effects seen in these studies were due to other developmental and/or systemic alterations.

Results from the first study indicate that OSMRβ KO mice exhibit AT inflammation, insulin resistance, increased hepatic and serum lipid concentrations, and beta cell hyperplasia on normal chow diet [52]. These results suggest that OSM signaling plays an important role in lipid handling in AT and liver. Also, wild-type B6 mice were injected with OSM twice daily for one week and glucose tolerance, insulin sensitivity, and ATM populations assessed. OSM treatment decreased the total number of F4/80+ cells, but the percentage of pro-inflammatory M1 ATMs was lower while the percentage of anti-inflammatory M2 ATMs increased [52]. Improvements in glucose tolerance and insulin sensitivity in OSM-treated mice were also observed, suggesting that possible metabolically favorable improvements likely have beneficial effects on lipid metabolism. However, it is difficult to assess whether the mice were healthy during the injections, as no food intake or body weight data were reported [52]. Possible decreases in appetite and body weight, resulting from the supraphysiological doses of OSM used in these experiments, could account for the alterations observed in glucose metabolism.

In a follow-up study by the same group, *ob/ob* mice were injected with OSM twice daily for one week [53]. Significant improvements in insulin sensitivity, glucose tolerance, AT inflammation, and hepatic steatosis were seen in OSM-injected mice. However, body weight also significantly decreased in the OSM-injected mice, and this may partially account for the improvements. Additionally, OSMRβ KO mice were fed a HFD for 8 weeks and exhibited greater body weight and food intake than control mice. Further, the insulin resistance, AT inflammation, and hepatic steatosis observed were more severe in the obese KO mice than in the control mice [53]. The severity of these effects was not resolved when OSMRβ KO mice were pair-fed with control mice, suggesting that the effects were independent of body weight gain. AT inflammation, ATM accumulation, and ATM polarization to the M1 phenotype was markedly increased in KO mice as early as 2 weeks on HFD. Hepatic lipid metabolism was more closely evaluated in this study, and OSMRβ KO mice demonstrated more severe liver lipid accumulation as determined by histology, tissue triglyceride quantification, and tissue total cholesterol quantification [53]. The OSMRβ KO mice also exhibited increased hepatic expression of fatty acid synthesis genes, which was not affected by pair feeding. These results suggest that altered lipid metabolism in the OSMRβ KO mice may be responsible, in part, for the insulin resistance reported in these animals.

While the results from both studies do support a putative role for OSM signaling in lipid metabolism in both AT and liver, the use of global OSMRβ KO mice makes it difficult to determine cause and effect and to assess whether their phenotype is related to developmental factors. Future studies should be conducted on tissue-specific or tissue-specific inducible OSMRβ KO mice to address current confounding data and the developmental and systemic effects of OSM.

## 7. Summary and Future Directions

Our understanding of OSM and its ability to modulate lipid metabolism is rudimentary. To date, there is confounding data on whether OSM induces metabolically beneficial [52,53] or metabolically detrimental effects [43,46,66]. Since OSM is highly produced in conditions of excess lipid storage as judged by adiposity [46], we predict that OSM will have profound effects on glucose and lipid metabolism (see Figure 1). As indicated above, it will be essential to examine tissue-specific manipulation of both OSM and OSMRβ to determine the primary tissue sources of OSM as well as the action of this cytokine on tissues involved in lipid metabolism.
